# Validation of a digital PCR method for quantification of DNA copy number concentrations by using a certified reference material

**DOI:** 10.1016/j.bdq.2016.08.002

**Published:** 2016-08-30

**Authors:** Liesbet Deprez, Philippe Corbisier, Anne-Marie Kortekaas, Stéphane Mazoua, Roxana Beaz Hidalgo, Stefanie Trapmann, Hendrik Emons

**Affiliations:** Directorate for Health, Consumers and Reference Materials, Joint Research Centre, European Commission, Retieseweg 111, 2440 Geel, Belgium

**Keywords:** Digital PCR, Method validation, Measurement uncertainty, Certified reference materials

## Abstract

Digital PCR has become the emerging technique for the sequence-specific detection and quantification of nucleic acids for various applications. During the past years, numerous reports on the development of new digital PCR methods have been published. Maturation of these developments into reliable analytical methods suitable for diagnostic or other routine testing purposes requires their validation for the intended use.

Here, the results of an in-house validation of a droplet digital PCR method are presented. This method is intended for the quantification of the absolute copy number concentration of a purified linearized plasmid in solution with a nucleic acid background. It has been investigated which factors within the measurement process have a significant effect on the measurement results, and the contribution to the overall measurement uncertainty has been estimated. A comprehensive overview is provided on all the aspects that should be investigated when performing an in-house method validation of a digital PCR method.

## 1 Introduction

Accurate quantification of the copy number concentration of specific nucleic acid sequences is important for several applications both within the fields of red biotechnology, (e.g. oncology and infectious diseases) and green biotechnology (e.g. GMO testing). During the last decade, digital PCR (dPCR) has shown to be the emerging technique for the sequence-specific detection and quantification of nucleic acids [1], [2]. The measurement principle of dPCR relies on partitioning the PCR mix across a large number of small individual reaction volumes, such that the distribution of the target sequence follows a binominal distribution function and that a part of the reaction volumes does not contain a copy of the target sequence [3]. Following an end-point PCR, partitions containing one or more copies of the target sequence are labelled positive and counted. The proportion of positive partitions is used to estimate the copy number concentration of the target sequence, taking into account the statistics of the binominal distribution [4]. Commercially available dPCR systems are based on two different approaches to partition the PCR mix: some use microfluidic chips on which the PCR mix is distributed over premanufactured chambers [5], [6] while others are based on oil-water emulsions to separate the solution into droplets [7], [8].

Digital PCR has the potential to replace quantitative real-time PCR (qPCR) for several of the current applications as it can have several advantages, including improved precision [9], reduced interference of PCR inhibitors [10] and independence of a calibration curve to determine the copy number concentration of the target sequence [11]. However, the measurement principle of the dPCR implies some essential prerequisites and failure to fulfil one or more of these, affects the reliability of the measured absolute copy number concentrations. First, the copies of the target sequence should be distributed over the partitions in a random and uniform manner meaning that there should be no aggregation of DNA sequences. Second, the volume of the partitions should be well-known and consistent within and between measurements. Third, partitions should be correctly classified as positive or negative after the end-point PCR [12].

Numerous reports on the development of new dPCR methods have been published during the past years. Maturation of these new developments into reliable analytical methods suitable for diagnostic or other routine testing purposes requires that the methods are validated for their intended use. Method validation is the tool to proof that a method is fit for purpose and to ensure that the measurement results are sufficiently reliable so that related decisions can be taken with confidence. International standards such as ISO/IEC 17025 [13] and ISO 15189 [14] also stress the need for method validation. There are several guidance documents on method validation [15], [16], [17] describing a series of tests that both verify the assumptions on which the analytical method is based and establish the performance characteristics of the method. Table 1 provides a list of performance characteristics that are typically assessed during method validation. Several of these performance characteristics are also included in the guidelines on Minimum Information for the publication of Quantitative dPCR Experiments (dMIQE) [18].

Here, a complete in-house validation is described for a dPCR method using the droplet digital™ PCR (ddPCR) system (Bio-Rad) which partitions the PCR mix in approximately 20,000 droplets with an individual volume < 1 nL. This ddPCR method amplifies a specific sequence of the human fusion transcript *BCR-ABL* and is intended to be used for the quantification of the absolute copy number concentration of a linearized plasmid carrying the *BCR-ABL* sequence in solution with a nucleic acid background. The approaches for method validation described in the following can be used as an example for the validation of other dPCR methods.

## 2 Materials and methods

### 2.1 Test material

The method validation was performed on samples of certified reference materials from the ERM‐AD623 set [19]. ERM-AD623 consists of 6 solutions of a double-stranded linearized plasmid carrying 3 DNA fragments specific for 3 human cDNA transcripts: the transcript of the breakpoint cluster region gene (*BCR*), the transcript of the glucuronidase beta gene (*GUSB*) and the aberrant transcript (*BCR-ABL* b3a2) consisting of a fusion of the *BCR* gene with the c-abl oncogene 1 (*ABL*). Each of the six solutions; ERM-AD623a, ERM-AD623b, ERM-AD623c, ERM-AD623d, ERM-AD623e and ERM-AD623f has a different certified copy number concentration: (1.08 × 10^6^ ± 0.13 × 10^6^), (1.08 × 10^5^ ± 0.11 × 10^5^), (1.03 × 10^4^ ± 0.10 × 10^4^), (1.02 × 10^3^ ± 0.09 × 10^3^), (1.04 × 10^2^ ± 0.10 × 10^2^) and (10.0 ± 1.5) copies(cp)/μL, respectively. The plasmid solutions were prepared in a T_1_E_0.01_ buffer (1 mM Tris, 0.01 mM EDTA, pH 8.0) supplemented with 50 mg/L of transfer RNA from *Escherichia coli* (*E. coli*). The certified copy number concentrations and the associated uncertainties assigned to the ERM-AD623 solutions were derived from measurement data of 3 metrology institutes using a chip-based dPCR technology (i.e. the BioMark™ system with 12.765 digital Arrays ™ from Fluidigm).

### 2.2 ddPCR method

The ddPCR method validated in this study targets a sequence specific for the human *BCR-ABL* transcript (referred here as the BCR-ABL ddPCR method). Also, a second ddPCR method was applied targeting a sequence specific for the *ABL* transcript (called the ABL ddPCR method). These ddPCR methods are based on two qPCR methods which were developed within the frame of a ‘Europe Against Cancer' program [20], [21]. The sequences of the primers and probes and their concentrations used in the ddPCR methods can be found in Supplementary data Table 1. The term ‘assay’ is used to refer to the combination of the specific primers and probes. All primers and probes were purified by HPLC (Life Technologies Europe BV). The PCR mix comprised 1 × ddPCR Supermix for Probes (Bio-Rad, cat no. 186-3010), suitable primers and probes, nuclease free water (Promega, cat no. P1193) and the DNA sample. To minimise the uncertainty from pipetting, all components, excluding the DNA sample, were premixed in the pre-sample mix, and the final PCR mix was prepared gravimetrically by combining the DNA sample with the pre-sample mix using a microbalance. The density of the pre-sample mix was determined by pipetting 100 μL on the microbalance using a calibrated pipette. The average density and the associated standard deviation (STD) of 10 replicate measurements were 1.0353 ± 0.0026 g/L.

Twenty microliters of the PCR mix were pipetted into the compartments of the Droplet Generator DG8™ Cartridge (Bio-Rad, 2 types were used: cat no. 186-3008 and 186-4008) and 70 μL of the Droplet Generation Oil for Probes (Bio-Rad, cat no. 186-3005) was added to the appropriate wells. The cartridges were covered with DG8™ Gaskets (Bio-Rad, cat no. 186-3009) and placed in a QX100™ Droplet Generator (Bio-Rad, cat no. 186-3002) to generate the droplets. Afterwards, the droplets were gently transferred to a semi-skirted and PCR clean 96-well PCR plate (Eppendorf, cat no. 0030 128.605) using a Pipet-lite ™XLS+ manual 8-channel pipette with the range 5–50 μL (Rainin, cat no. L8-50XLS+). The PCR plate was sealed with pierceable foil (Bio-Rad, cat no. 181-4040) using a PX1™ PCR Plate Sealer (Bio-Rad, cat no. 181-4000). After sealing, the PCR plate was placed in a C1000 Touch™ Thermal Cycler (Bio-Rad, cat no. 185-1197) for PCR amplification. The PCR protocol can be found in the Supplementary data Table 2. The droplet reading was done with the QX 100 Droplet reader (Bio-Rad, cat no. 186-3001) using ddPCR™ Droplet Reader Oil (Bio-Rad, cat no. 186-3004).

### 2.3 Data analysis

Data acquisition and analysis were performed with the software package QuantaSoft (Bio-Rad). As measurements were spread over an extended period, three different versions of this software were used: version 1.3.2.0, version 1.6 and version 1.7.4. The fluorescence amplitude threshold, distinguishing the positive from the negative droplets was set manually by the analyst as the midpoint between the average fluorescence amplitude of the positive and negative droplet cluster. The same threshold was applied to all the wells of one PCR plate. Measurement results of single PCR wells were excluded on the basis of technical reasons in case that (i) the total number of accepted droplets was <10,000, (ii) the average fluorescence amplitudes of positive or negative droplets were clearly different from those of the other wells on the plate, or (iii) 5 % of the accepted droplets had a fluorescence amplitude significantly below the average amplitude of the negative droplet cluster (i.e. average − 4 × STD). The average number of accepted droplets of the valid measurement results was around 17,000.

The numbers of positive and accepted droplets were transferred to an in-house developed spread sheet to calculate the copy number concentration in the sample ($c_{sample}$) using Eq. (1) with a droplet volume set at 0.834 nL [22].

(1) $$c_{sample}=Df_{sample}\times Df_{PCR}\times \left(\frac{1}{A\times V_{d}}\right)\times \frac{\left(\log\left(1-\frac{P}{A}\right)\right)}{\left(\log\left(1-\frac{1}{A}\right)\right)}$$

With *Df_sample_*: dilution factor of the DNA sample before adding to the PCR mix;*Df_PCR_*: dilution factor of the DNA solution in the PCR mix;*A*: number of analysed droplets;*P*: number of positive droplets;$V_{d}$: droplet volume.

Throughout this manuscript, the term sample copy number concentration$\left(c_{sample}\right)$ is used to describe the copy number concentration of the undiluted sample, while the term PCR copy number concentration ($c_{PCR}$) is used to refer to the copy number concentration in the PCR mix.

The dMIQE checklist [18] of these ddPCR experiments can be found in the Supplementary data Table 3.

## 3 Results

### 3.1 Selectivity

The primers and probes of the BCR-ABL ddPCR method are also used in a standardised qPCR method developed during a large inter-laboratory study and the absence of nonspecific amplification artefacts in qPCR has been shown [20], [21]. The selectivity of the BCR-ABL ddPCR method was experimentally assessed by performing 4 replicate measurements of a matrix blank consisting of 1 × T_1_E_0.01_ buffer with the nucleic acid background of the ERM-AD623 samples (i.e. transfer RNA from *E. coli*) and 4 replicates of a positive control consisting of an undiluted sample of ERM-AD623a at a PCR copy number concentration of 54000 cp/μL. Results showed a clear difference in fluorescence amplitude between the negative droplet cluster (average 1764 and STD 135) and the positive droplet cluster (average 5418 and STD 212). With the threshold placed at the midpoint between the average fluorescence amplitudes of the positive and negative droplet cluster, no droplets in matrix blank replicates were classified as positive (0/61275) and only 0.057 % (33/57895) of the droplets in the positive control replicates were classified as negative.

### 3.2 Working range

The working range of the BCR-ABL ddPCR method was investigated by measuring one sample of each of the five lowest ERM-AD623 concentration levels at different PCR copy number concentrations: ERM-AD623b was measured at 5400 cp/μL, ERM-AD623c at 2575 cp/μL, ERM-AD623d at 255 cp/μL, ERM-AD623e at 26 cp/μL and ERM-AD623f at 2.5 cp/μL. For each concentration, 8 replicate measurements were performed, and the replicates were spread over 4–5 cartridges and randomly positioned on the 96-well PCR plate.

**Fig. 1:**
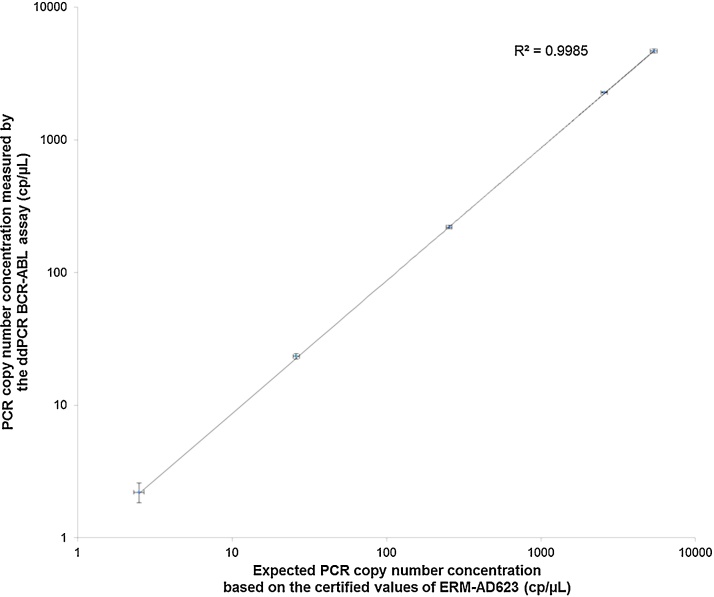
Linearity of the BCR-ABL ddPCR method when measuring ERM-AD623 samples within a PCR copy number concentration range of 2.5 cp/μL to 5400 cp/μL. The data points represent the average result for eight replicate measurements, and the vertical error bars represent the associated STD. The horizontal error bars represent the standard uncertainty associated with the certified values of the ERM-AD623 samples.

None of the measurement results was rejected based on the technical reason exclusion criteria described in Section 2.3. The relative STD of the replicate measurement results was < 5% for the PCR copy number concentrations between 26 and 5400 cp/μL. At the lowest PCR copy number concentration of 2.5 cp/μL, the relative STD increased to 16.9 % suggesting that this concentration might be out of the working range. A precise determination of the lower end of the working range is discussed during the assessment of the limit of quantification (LOQ) of the method in Section 3.6. The relation between the expected PCR copy number concentration $\left(c_{PCR,exp}\right)$ and the measured PCR copy number concentration ($c_{PCR,meas}$) was linear (r^2^ = 0.9985, see Fig. 1) and the equation of the regression line was $c_{PCR,meas}=0.8677\times c_{PCR,exp}$. This regression line indicates that $c_{PCR,meas}$ is about 13 % lower than the $c_{PCR,exp}$ suggesting a bias between the certified copy number concentrations of ERM-AD623 and the copy number concentrations measured by the BCR-ABL ddPCR method. A more precise estimate of this bias based on many measurement results was obtained during the assessment of the method accuracy below.

### 3.3 Accuracy

The five highest concentration levels of ERM-AD623 were measured with the BCR-ABL ddPCR method at PCR copy number concentrations of 250–450 cp/μL (for ERM-AD623a, b, c and d) and 25–35 cp/μL (for ERM-AD623e). The samples of ERM-AD623a, ERM-AD623b and ERM‐AD623c were gravimetrically diluted in T_1_E_0.01_ buffer to a nominal concentration between 1000 cp/μL and 1800 cp/μL before adding to the PCR Mix. The experiments were performed in 3 runs and each ERM‐AD623 concentration level was measured with 12 replicates in runs 1 and 3, and 16 replicates in run 2. The replicate measurements within one run were carried out under repeatability conditions meaning: the same analyst, the same pre-sample mix, cartridges from the same batch, the same instruments and randomly positioned on the same 96-well PCR plate. Between the runs, intermediate precision conditions were applicable, meaning: 3 different analysts, 2 different droplet generators, 2 different droplet readers, 2 different types of cartridges, 3 different batches of reagents and 3 different versions of the QuantaSoft software. In total, 200 measurement results were obtained, and only 4 of them were rejected because of technical reasons.

The nested design of this experiment allowed an estimation of the method repeatability and the run-to-run variation as prescribed by ISO 5725-3. [23] The results were grouped per run and analysed with a one-way analysis of variance (ANOVA) test. For each ERM-AD623 concentration level, the relative repeatability $\left(s_{repeat,rel}\right)$ and the relative run-to-run variation $\left(s_{run,rel}\right)$ both expressed as STD were calculated using Eqs. (2) and (3), respectively.

(2) $$s_{repeat,rel}=\frac{\sqrt{MS_{withinrun}}}{{\bar{c}}_{sample,meas}}$$

(3) $$s_{run,rel}=\frac{\sqrt{\frac{MS_{betweenrun}-MS_{withinrun}}{{\bar{n}}_{repli}}}}{{\bar{c}}_{sample,meas}}$$

With $MS_{\mathrm{within}\;\;\mathrm{run}}$: the within run mean of squares calculated by one-way ANOVA${MS}_{between\;\;\;run}$: the between run mean of squares calculated by one-way ANOVA${\bar{n}}_{\mathrm{repli}}$: average number of replicates per run${\bar{c}}_{sample,meas}$: average measured sample copy number concentration over all runs.

It should be noted that $s_{repeat,rel}$ and $s_{run,rel}$ are estimates of the true STD and are subject to random fluctuations. It can, therefore, happen that $MS_{betweenrun}$ is smaller than $MS_{withinrun}$ and then $s_{run,rel}$ cannot be estimated with Eq. (3). In this case, we considered $s_{run,rel}$ equal to zero as it is negligible compared to the $s_{repeat,rel}$.

Based on the $s_{repeat,rel}$ and $s_{run,rel}$ the relative standard uncertainty of the method precision ($u_{precision,rel}$) associated with the average measured sample copy number concentration (${\bar{c}}_{sample,meas}$) was calculated using Eq. (4).

(4) $$u_{\mathrm{precision},rel}=\sqrt{\frac{s_{\mathrm{repeat},\mathrm{rel}}^{2}}{{\bar{n}}_{\mathrm{repli}}\times n_{\mathrm{run}}}+\frac{s_{\mathrm{run},\mathrm{rel}}^{2}}{n_{\mathrm{run}}}}$$

With $n_{run}$: number of runs, which is 3 in this case

The calculated values for $s_{repeat,rel}$, $s_{run,rel}$, and $u_{precision,rel}$ are shown in Table 2. The five values obtained for each parameter (one per ERM-AD623 concentration level) were combined into one pooled value by taking the root mean square (RMS, also called quadratic mean) calculated as the square root of the average of the squared values. The pooled relative repeatability ($s_{repeat,pooled,rel}$) was 6.1 %, the pooled relative run-to-run variation ($s_{run,pooled,rel}$) was 2.9 % and the pooled relative standard uncertainty related to precision ($u_{precision,pooled,rel}$) was 1.9 %.

The trueness of the BCR-ABL ddPCR method was evaluated by estimating the relative bias ($bias_{rel})$ for each ERM-AD623 concentration level as the relative difference between the average measured sample copy number concentration (${\bar{c}}_{sample,meas}$) and the certified sample copy number concentration ($c_{sample,cert})$(see Eq. (5)).

(5) $$\mathrm{bia}s_{\mathrm{rel}}=\frac{{\bar{c}}_{\mathrm{sample},\mathrm{meas}}-c_{\mathrm{sample},\mathrm{cert}}}{c_{\mathrm{sample},\mathrm{cert}}}$$

The average relative bias (${\bar{bias}}_{rel}$), calculated as the arithmetic mean of the five values for $bias_{rel}$(one per ERM-AD623 concentration level), was −9.6 %. To evaluate whether or not this ${\bar{bias}}_{rel}$ is significant, the uncertainty associated with this bias estimate was calculated taking into account the uncertainty associated with the average measured copy number concentrations (i.e. $u_{precision,pooled,rel}$) and the relative standard uncertainty associated with the certified copy number concentration of each ERM-AD623 concentration level ($u_{cert,rel}$). Both uncertainty contributions were combined in the relative uncertainty of the bias estimate ($u_{bias,rel}$) using Eq. (6).

(6) $$u_{bias,rel}=\sqrt{{u_{precision,pooled,rel}}^{2}+\frac{\overset{}{\underset{}{\sum_{i=a}^{e}}}{{(u}_{cert,rel,i})}^{2}}{n_{cert}}}$$

With $n_{cert}$ : the number of certified reference materials used in the assessment of the bias.

The $u_{bias,rel}$ was 5.4 %, and the relative expanded uncertainty of the bias estimate ($U_{bias,rel}$) was calculated to be 10.9 % using Eq. (7).

(7) $$U_{bias,rel}=2\times u_{bias,rel}$$

As the absolute value of the estimated ${\bar{bias}}_{rel}$ is smaller than $U_{bias,rel}$ this bias cannot be considered significant, but there is a strong indication that the BCR-ABL ddPCR method has the tendency to measure lower copy number concentrations than the chip-based dPCR method used for the certification of the copy number concentration of the ERM-AD623 solutions.

### 3.4 Measurement uncertainty

**Fig. 2:**
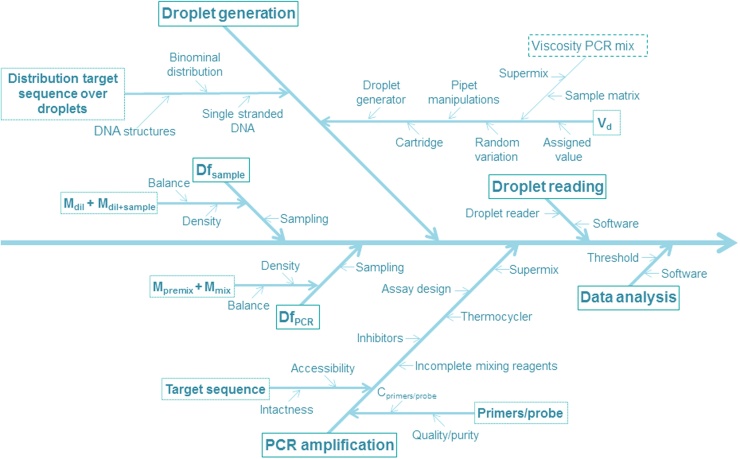
A schematic overview of all factors which may contribute to the uncertainty of the measurement results obtained with BCR-ABL ddPCR method as performed in this validation study. *C_primers/probe_*: concentration primers and probe, *Df_sample_*: dilution factor of sample before addition to PCR mix, *Df_PCR_*: dilution factor of sample in the PCR mix, *M_dil_*: mass of diluent, *M_dil+sample_*: mass of diluent and sample, *M_premix_*: mass of pre sample mix, *M_mix_*: mass of the PCR mix with sample, *V_d_*_:_ volume of the droplets

Measurement uncertainty may arise from many sources and a complete list of all potential sources is a good starting point for a comprehensive estimate of the overall measurement uncertainty [24]. Fig. 2 gives a schematic overview of all factors which may contribute to the uncertainty of the measurement results obtained with the BCR-ABL ddPCR method as performed here.

The results from the assessment for the method precision provided an estimate of the contribution of several uncertainty sources. The uncertainty contributions of the random effects, including sampling, random variation in the droplet volume, binominal distribution and the position in the thermocycler, were included in the $s_{repeat,pooled,rel}$ while the $s_{run,pooled,rel}$ covers the uncertainty arising from the run-to-run effects such as the type of cartridges, the reagent batches, the droplet reader/generator and the analyst.

The tendency to measure with the BCR-ABL ddPCR method lower copy number concentrations than the certified copy number concentrations of the ERM-AD623 samples indicates that some of the remaining sources also have an important effect on the measurement result and make a significant contribution to the overall measurement uncertainty. These factors were therefore investigated in greater detail. The estimation of the uncertainty contribution of several individual factors was based on previous knowledge and uncertainty components < 1 % were not considered as significant. These negligible uncertainty sources are the accuracy of the weighing, the uncertainty associated with density determination of the pre-sample mix and the uncertainty related to the purity and quality of the HPLC-purified primers and probes [25]. The samples that are intended to be measured with the BCR-ABL ddPCR method are highly purified solutions of linearized plasmid DNA in a T_1_E_0.01_ buffer with a nucleic acid background. As these solutions are candidate certified reference materials, the intactness of the DNA molecules and their stability has already been investigated. Due to the particular nature of the samples, the following sources of uncertainty were also considered to be negligible: presence of single-stranded DNA, presence of PCR inhibitors, the presence of secondary DNA structures, which might disturb the random distribution of the target sequence over the droplets, and the accessibility and intactness of the target sequence.

The droplet volume determines the absolute copy number concentration calculated with Eq. (1). We have used a droplet volume of 0.834 nL as this volume was previously measured in our laboratory using the same equipment, the same type of supermix and the same type of samples. The relative standard uncertainty associated with the measured droplet volume ($u_{V_{d}},rel$) was 1.8 % [22].

Two sources of measurement uncertainty (i.e. the assay and the threshold setting) were investigated in a dedicated study to estimate their contribution to the overall measurement uncertainty.

#### 3.4.1 Uncertainty component related to the assay

By measuring the ERM-AD623 samples with another combination of primers and probe, it has been investigated if the assay itself has a significant contribution to the measurement uncertainty. Therefore, the five highest concentration levels of ERM-AD623 were also measured with the ABL ddPCR method. The set-up of the experiments was identical to the experiments performed to assess the accuracy of the BCR-ABL ddPCR method, meaning 3 runs with each 12–16 replicates per ERM-AD623 concentration level under repeatability conditions within the runs and intermediate precision conditions between the runs. Fourteen of the 200 measurement results obtained with the ABL ddPCR method were rejected because of technical reasons. The results of the ABL and the BCR-ABL assay were grouped per assay and per ERM-AD623 concentration level. For each concentration, the relative STD due to the assay ($s_{assay}$) was calculated using one way-ANOVA and Eq. (8).

(8) $$s_{assay,rel}=\frac{\sqrt{\frac{MS_{betweenassay}-MS_{withinassay}}{{\bar{n}}_{meas,assay}}}}{{\bar{c}}_{sample,meas}}$$

With $MS_{\mathrm{within}\;\;\;\;\;\mathrm{assay}}$: the mean of square of results obtained with one assay${MS}_{between\;assay}$: the mean square between results obtained with the two assays${\bar{n}}_{\mathrm{meas},\mathrm{assay}}$: the average number of measurements per assay${\bar{c}}_{sample,meas}$: average measured sample copy number concentration from both assays

The five values for $s_{assay}$ (one per ERM-AD623 concentration level) were pooled by calculating the RMS. The resulting $s_{assay,pooled,rel}$ was 1.0 % indicating that the uncertainty contribution of the assay can be considered as negligible.

#### 3.4.2 Uncertainty related to the threshold setting

The classification of the droplets into positive or negative depends on the fluorescence amplitude of the threshold. For the experiments performed here, the threshold was set at the midpoint between the average fluorescence amplitude of the positive and negative droplet cluster. However, other approaches can be used, and they may lead to different measurement results. The variability among the results obtained with different threshold settings is caused by the presence of droplets with fluorescence amplitude above the upper boundary of the negative cluster and below the lower boundary of the positive cluster, the so-called rain droplets. We defined the boundaries of the negative and positive droplet cluster as the average amplitude ± 4 × STD as this range would theoretically include all droplets of that cluster in case of a normal distribution of the fluorescence data. It is unclear whether or not the rain droplets, in reality, contain a copy of the target sequence as we observed rain droplets in both the matrix blank and the highly positive control sample. An estimation of the maximum uncertainty contribution related to the threshold setting can be obtained by analysing the same measurement data with 3 completely different approaches to classifying the rain droplets:

• Low threshold placed at the upper boundary of the negative droplet cluster (all rain droplets are considered positive)
• High threshold placed at the lower boundary of the positive droplet cluster (all rain droplets are considered negative)
• Rain removal: rain droplets were not considered as accepted droplets [26]

The impact of small changes in the ratio of positive droplets/accepted droplets on the measured value depends on the PCR copy number concentration at which the measurements are performed: the effect is larger at the lower and the higher end of the working range. The effect of the threshold setting was therefore investigated at 5 different PCR copy number concentrations. The data of the experiment performed to determine the working range (Section 3.2.) were reused for this purpose. The original results (obtained with a threshold placed at the midpoint) and these reanalysed results were grouped per replicate measurement. One-way ANOVA and Eq. (9) were used to calculate the relative STD for the results obtained with the different threshold settings ($s_{thres,rel}$) (see Table 3).

(9) $$s_{thres,rel}=\frac{\sqrt{MS_{withinrepli}}}{{\bar{c}}_{PCR,meas}}$$

With ${MS}_{within\;repli}$: the mean of squares of the results for one replicate measurement${\bar{c}}_{PCR,\mathrm{meas}}$: the average measured PCR copy number concentration

These results show that the maximum uncertainty related to the threshold setting can be considered negligible for measurements performed with the BCR-ABL ddPCR method in the PCR copy number concentration range between 26 and 2575 cp/μL.

#### 3.4.3 Overall measurement uncertainty

The uncertainty contributions found to be significant can be combined into one relative expanded measurement uncertainty ($U_{meas,rel})$ using Eq. (10). This equation can be used to estimate the *U_meas,rel_* for any measurement result obtained with the BCR-ABL ddPCR method provided that the measured sample is similar to the ERM-AD623 samples and that the PCR copy number concentration is in the range of 25–450 cp/μL.

(10) $$U_{meas,rel}=2\times \sqrt{\frac{{s_{repeat,pooled,rel}}^{2}}{n_{meas}}+{\frac{s_{run,pooled,rel}}{n_{run}}}^{2}+{u_{V_{d},rel}}^{2}+{u_{bias,rel}}^{2}}$$

With $n_{meas}$: the number of measurements on which the measurement result is based$n_{run}$: the number of runs over which the measurements are spread

For a measurement result obtained as the average of 4 replicate measurements performed in a single run the *U_meas,rel_* is 14.2 %.

### 3.5 Limit of detection (LOD)

The LOD is defined as the lowest PCR copy number concentration that can be distinguished from zero with a level of confidence of 95 %. A rough estimate of the LOD of the BCR-ABL ddPCR method was obtained by measuring one sample of ERM-AD623f in 64 replicates at a concentration of 0.50 cp/μL in the PCR mix. The replicate measurements were performed under repeatability conditions. Four of the measurement results were rejected because of technical reasons and all of the 60 valid replicate measurements gave a positive result. The average measured PCR copy number concentration was 0.56 cp/μL with a relative STD of 34.4 %. These results indicate that the LOD of this method is < 0.50 cp/μL in the PCR mix.

### 3.6 Limit of quantification (LOQ)

The LOQ is defined as the lowest PCR copy number concentration for which the method provides results with an acceptable uncertainty. So, the LOQ of a method depends on the level of uncertainty considered acceptable given the intended use of the method. For the purpose of certifying the absolute copy number concentration of purified plasmid solutions, we considered the maximum acceptable expanded measurement uncertainty to be 30 % for a measurement result obtained as an average value of four replicate measurements. This maximum acceptable expanded measurement uncertainty is 2 times larger than the expanded uncertainty obtained for measurements with the PCR copy number concentration range of 25–450 cp/μL, but at very low concentrations, stochastic effects will have a major impact on the method repeatability and therefore on the overall measurement uncertainty.

Samples of ERM-AD623f were measured with the BCR-ABL ddPCR method at a PCR copy number concentration of 3.50 cp/μL. Measurements were spread over two runs, and one run consisted of 12 replicate measurements. Within one run repeatability conditions were applicable and between the runs intermediation precision conditions were used as described before.

In total, 24 measurement results were obtained, and only one was rejected because of technical reasons. The average measured PCR copy number concentration was 3.35 cp/μL, and the relative bias between the measured value and the certified value was − 4.3 % (according to Eq. (5)). The results were grouped per run and analysed with ANOVA to estimate the measurement precision of the BCR-ABL ddPCR method. Using Eqs. (2) and (3), the relative repeatability ($s_{repeat,rel})$ was calculated to be 17.0 % and the run-to-run variation ($s_{run,rel})$ was considered negligible as $MS_{betweenrun}$ < $MS_{withinrun}$.The relative standard uncertainty related to precision ($u_{precision,rel}$) was estimated to be 5.0 % with Eq. (4). The expanded uncertainty associated with the bias estimate ($U_{bias,rel})$ was 18.0 % (according to Eq. (7)), indicating that the relative bias of −4.3 % is not significant.

To calculate the overall measurement uncertainty for an average measurement result of 4 replicates one has to add the estimate of the uncertainty contribution from the threshold setting $\left(s_{threshold}\right)$ to Eq. (10) as this uncertainty is not negligible at the limits of the working range (see Table 3).

The overall measurement uncertainty was calculated to be 28.9 % for an average measurement result from 4 replicates indicating that a PCR copy number concentration of 3.50 cp/μL is a good estimate of the LOQ for the intended use of the method.

### 3.7 Robustness (ruggedness)

During the robustness test, the effect of small deviations in relevant method parameters on the method performance and the measurement results are investigated. For the BCR-ABL ddPCR method, relevant method parameters that are likely to influence the method outcome are the primer and probe concentrations and the annealing temperature.

The effect of minor variations in the primer and probe concentrations was investigated by performing the BCR-ABL ddPCR method with three different concentrations: the optimal concentrations as described in Supplementary data Table 1, concentrations that are 10 % lower and concentrations that are 10 % higher than the optimal concentrations. For each primer and probe concentration level, 10 replicate measurements were performed for one sample of ERM-AD623d at an expected PCR copy number concentration of 310 cp/μL. Three measurement results were rejected because of technical reasons. To test the effect of small deviations in the annealing temperature, the BCR-ABL ddPCR was performed at three different annealing temperatures: 60 °C (the optimal annealing temperature) 61 °C and 59 °C. For each annealing temperature, 14 replicate measurements of one ERM-AD623c sample were done at an expected PCR concentration of 3200 cp/μL. One measurement result was rejected because of technical reasons.

The results of the robustness assessment are shown in Table 4. They indicate that minor deviations of the optimal primers/probe concentration and the annealing temperature do not have a significant effect on the obtained measurement results taking into account the associated measurement uncertainty.

## 4 Discussion

The approaches used in this validation of the BCR-ABL ddPCR method are based on the recommendations described in various guidance documents on method validation and estimation of the measurement uncertainty [15], [16], [24] and the study design was adapted to the intended use of the method and the availability of certified reference materials. It should also be noted that this method validation covers only the performance parameters of the dPCR method itself. There are several additional factors influencing the value of dPCR measurement results for diagnostics and other decisions such as the biological variability of the target sequence, the sample source, the sample preparation and the sample storage [27], [28].

The method validation study described here may not be suitable for each dPCR method, however, there are some general considerations which are applicable for any dPCR method validation.

### 4.1 Selectivity

Analytical selectivity is defined as the degree to which the method can quantify the particular analyte accurately without the interference of other substances which could be present in the samples. The interfering substances may cause a bias by increasing or decreasing the signal attributed to the analyte. For dPCR, selectivity can be translated into the degree to which partitions classified as positive contain one or more copies of the target sequence, and the negative partitions contain no copy of the target sequence. Interferences could cause non-specific amplification or PCR inhibition. The development of a dPCR method with a good selectivity requires a conscious design of the PCR assay including a blast search for similar sequences and a thorough optimisation of the primer/probe concentrations and the annealing temperature. During method validation, the selectivity should be experimentally assessed by measuring the target sequence in samples to which interferences possibly present in real-life samples are deliberately introduced [15]. Examples of these interfering substances are highly similar sequences or organic substances such as phenol or ethanol introduced during the nucleic acid extraction. Analysis of a matrix blank, i.e. a sample with the same background and interfering substances as a real sample but without the target sequence can be used to identify interfering substances which lead to false positive partitions. A positive control sample with a high concentration of the target sequence can be analysed to identify interfering substances leading to false negative partitions. These positive control samples are preferably routine test samples, but in case that these are not available, spiked samples, in which the target sequence is added at high concentration, could be used as an alternative.

### 4.2 Working range

**Fig. 3:**
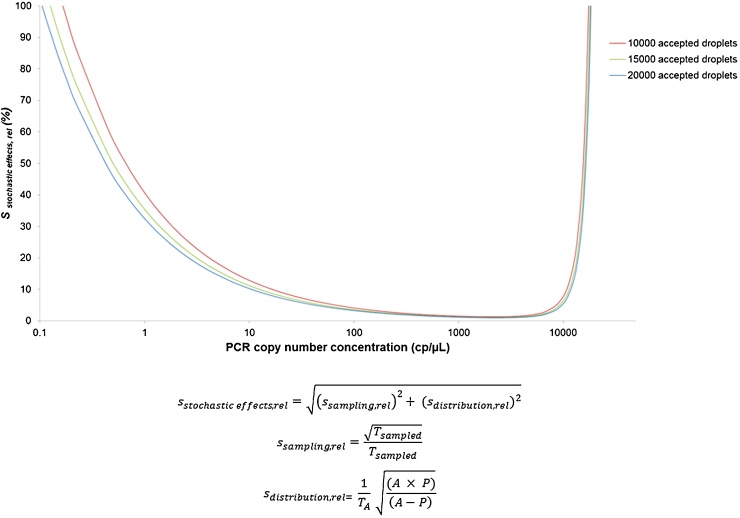
The relative STD caused by stochastic effects in relation to the PCR copy number concentration for the ddPCR system. The relative STD resulting from stochastic effects ($s_{stochastic\;\;\;\;\;\;\;\;\;\;\;effects,rel}$) consists of two components: the relative STD caused by the stochastic effects when sampling the DNA solution added to the PCR mix ($s_{sampling,rel}$) and the relative standard deviation caused by the stochastic effects of the distribution of the target sequence over the analysed droplets ($s_{distribution,rel}$). The estimation of the $s_{sampling,rel}$ is based on the Poisson distribution, and the estimation of $s_{distribution,rel}$ is based on the binominal distribution as described in [29]. $T_{sampled}$: the expected number of target sequences sampled, $T_{A}$: the expected number of target sequences in the analysed droplets, A: number of analysed droplets, P: number of positive droplets.

Digital PCR systems can detect a wide range of copy number concentrations ranging from one single copy to several hundreds or thousands of copies depending on the number of analysed partitions. However, the precision (and, therefore, also the reliability) of measurement results is not constant across this whole range due to stochastic effects which have an important influence at the lower and upper limit of the range [5], [29]. Stochastic effects mainly play a role during two steps of the dPCR measurement procedure: the sampling of the DNA solution added to the PCR mix and the distribution of the target sequence over the analysed partitions. Fig. 3 shows the relationship between the theoretical relative STD caused by these stochastic effects and the PCR copy number concentration for the ddPCR system.

### 4.3 Measurement precision

Precision is a measure of the variability in independent measurement results obtained for the same sample under stipulated conditions. Depending on the stipulated conditions, measurement precision can be divided into method repeatability, intermediate precision and reproducibility. Within the frame of a single laboratory validation, both the method repeatability and intermediate precision need to be investigated. Repeatability is a parameter for the variability in results of measurements performed by a single analyst using the same equipment and reagents during a short period of time. Intermediate precision gives an estimate of the variation in results from measurements made under conditions which are more variable than repeatability conditions. Ideally, the effect of all sources of variation that could occur during routine use in a single laboratory should be investigated. The assessment of method reproducibility requires measurement results obtained by different laboratories. This information is quite valuable but not mandatory in case of a single laboratory method validation [15].

### 4.4 Trueness

Measurement trueness is an expression of how close the mean of an infinite number (i.e. a large number in reality) of results produced by the method comes to a reference value. There are three general approaches to obtain a suitable reference value: i) use of certified reference materials, ii) recovery experiments using spiked samples, and iii) comparison with results obtained from another method. For the first option, it is important that the chosen certified reference material is appropriate, meaning the same or a very similar matrix and a target sequence copy number concentration within the same range as the routine samples [15]. In case no suitable certified reference material is available, it is possible to use spiked samples by adding a known copy number concentration of the target sequence in a matrix blank. However, one should note that these samples could stipulate an over-optimistic trueness assessment as the spiked target sequences might be easier accessible for amplification or more intact than the normal target sequence in a routine sample. It is also possible to assess the trueness by comparing results from the candidate method with those obtained from an alternative validated method. There are only a few methods that can verify the absolute copy number concentration measured by dPCR. One option is to compare with the results obtained with UV spectrophotometric methods that rely on the molar absorbance of the nucleic acids present in the solution [11], [30]. However, this is only possible for a limited number of sample types which are pure solutions of a single well-defined nucleic acid sequence. One additional drawback is the need for a series of carefully prepared gravimetrical dilutions as the optimal concentration range for UV spectrometric methods is usually several magnitudes larger than the optimal concentration range for dPCR measurements. A comparison with other sequence-specific quantification methods is also complicated. Quantitative real-time PCR results rely on the copy number concentration assigned to the calibrant. The use of another dPCR system, preferably one that uses a different technique to partition the PCR mix could be an option. In any case, the alternative method has to be validated and its measurement uncertainty must be correctly estimated.

### 4.5 Measurement uncertainty

Measurement uncertainty may arise from many sources, and these sources of uncertainty can be divided into five different levels: random effects, run-to-run effects, laboratory bias, method bias and matrix variation effects [16]. The uncertainty contribution of the random effects and run specific effects are assessed as method repeatability and run-to-run variation, respectively. The laboratory bias can be estimated from the method reproducibility obtained in large collaborative trails. In case of single-laboratory validation a trueness test can be used to assess the combination of the laboratory and method bias [16].

Typically, the individual sources of uncertainty are only investigated when they might be significant compared to the uncertainty associated with the precision or the bias estimate of the method. Uncertainty contributions that are smaller than 1/3 of the largest uncertainty component will have no significant effect on the overall measurement uncertainty and can be considered negligible [24]. Two sources of measurement uncertainty that are specific for dPCR measurements are discussed in greater detail in the following: the uncertainty contribution of the threshold setting and the uncertainty contribution of the assigned partition volume.

The importance of the uncertainty contribution of the threshold setting is determined by the number of partitions with intermediate fluorescent amplitude (meaning between the fluorescent amplitude of the negative and the positive partitions). The causes of this intermediate fluorescent amplitude are multiple, including abnormal sized partitions, presence of PCR inhibitors, reduced accessibility of the target sequence, non-specific amplification and incomplete mixing of PCR reagents. During the method development, efforts should be made to reduce the amount of partitions with intermediate fluorescent amplitude to a minimum by a good selection of the primer and probe sequences, careful titration of primer and probe concentrations and optimisation of the PCR conditions. Improving the quality of the analysed samples might also have an effect. During the method validation, the amount of partitions with intermediate fluorescent amplitude should be quantified both in samples with a high and a low copy number concentration of the target sequence to get information about their proportion compared to the positive and negative partitions, respectively. Supplementary data Fig. 1 shows the results of a theoretical simulation for the ddPCR system in which the maximum uncertainty related to the threshold setting is calculated for different proportions of rain droplets. In case of very low proportions of rain droplets (e.g. 0.01 % of the negative droplets or 0.1 % of the positive droplets) the maximum uncertainty related to the threshold setting can be considered negligible compared to the other uncertainty contributions. However, for ddPCR methods with higher proportions of rain droplets the threshold-related uncertainty might become a significant contributor to the overall measurement uncertainty. The uncertainty related to the threshold setting is not constant over the whole working range of a dPCR method and will be larger at the limits.

The uncertainty of the partition volume contributes to the overall measurement uncertainty of a dPCR measurement when absolute copy number concentrations are measured. The manufacturer often provides the partition volume of a dPCR system without any information about the associated uncertainty. Results of independent attempts to verify the partition volume and to estimate the associated uncertainty have been described for different dPCR systems [22], [29], [31], [32]. The obtained results do not always agree, and more research is still needed. The uncertainty on the partition volume is probably one of the major reasons of the measurement bias between different dPCR systems as also observed during the method validation presented above. In the case of the ddPCR system, the volume of the droplets could be influenced by the type of samples, the cartridges and the instruments, but especially by the type of supermix, which plays a major role [32]. Therefore it is important to use the droplet volume which is the most appropriate for the specific ddPCR method and to include the uncertainty associated with this droplet volume in the overall measurement uncertainty budget.

### 4.6 Limit of detection and quantification

Many of the guidance documents on method validation describe statistical approaches for the determination of LOD and LOQ that depend on the assumption of normal distribution [16]. The measurement principle of the dPCR makes it possible to detect up to one copy of a target sequence and the Poison distribution is applicable at these very low copy number concentrations. Therefore it is more reasonable to assess the LOD and LOQ by performing many replicate measurements at very low copy number concentrations. The assessment should start by clearly defining the level of confidence appropriate for the intended use of the method. In case that the copy number concentration of the target sequence in the routine samples will be always well above the LOD, it is sufficient to have a rough estimate of the LOD as done in the method validation presented above. When performing an experiment with 60 replicate measurements exactly at the LOD there would be on average 3 negative measurement results, as this represents 5 % of the cases. If all of the 60 replicate measurements performed at a certain PCR copy number concentration are positive, it can be reasonably assumed that this concentration is above the LOD of the method. More accurate estimates of the LOD are required for dPCR methods that are intended to be used to measure samples in which the target sequence could be absent, and this absence would lead to relevant decisions. In this case, negative measurement results should be reported as copy number concentration < LOD with a specified confidence level.

The LOD and LOQ of a dPCR method depend on the number of analysed partitions and the total volume of the analysed partitions. In case of the ddPCR system the number of analysed partitions varies. The impact on the LOD and LOQ can be illustrated by calculating the theoretical minimum LOD based on the Poisson distribution, both at the level of sampling and the distribution of the target sequence over the droplets. In the case of 15 000 analysed droplets with a droplet volume of 0.834 nL, the minimum theoretical LOD will be 0.32 cp/μL in the PCR mix, as 5 % of the measurements at this level will not have a single copy of the target sequence in the analysed droplets. In case that the number of analysed droplets is 10 000, the minimum theoretical LOD will increase to 0.44 cp/μL. Careful manipulation of the droplets and rejection of measurement results with a low number of accepted droplets (as done in this study by applying the technical reason exclusion criteria mentioned in Section 2.3) are therefore required to guarantee a certain LOD and LOQ.

### 4.7 Robustness

The robustness of a method is tested by making small but deliberate changes to the method variables and studying the effect on the method performance [16]. The investigated method variables should be expected to have an important influence on the method performance, i.e. the measurement result, and the size of the deliberate changes should be relevant for the routine use of the method. For a dPCR method, the effects of small changes in the primer and probe concentrations and the annealing temperature are useful to investigate as they can occur due to random pipetting errors and temperature fluctuations in the thermocycler.

## 5 Conclusion

The method validation described here can be used as an example for other single laboratory validations of dPCR methods. However, the extensiveness of a method validation should always depend on the intended use of the method and on the acceptable level of measurement uncertainty. The experiments should be conducted in a manner which provides a realistic view of all the factors possibly affecting the measurement result during routine use of the method, as well as covering the concentration ranges and sample types within the scope of the method [24]. The most challenging part of the validation of dPCR methods is probably the verification of the trueness, as representative samples with a reference value are often difficult to find. The development of suitable certified reference materials will diminish this problem and can promote the transition of newly developed dPCR methods into reliable analytical methods suitable for diagnostic or other routine testing purposes.

## Figures and Tables

**Table 1:** Critical performance characteristics which should be assessed during the validation of a quantitative analytical method.

Performance characteristic	Description
Selectivity	Degree to which the method can quantify the particular analyte (i.e. a specific target sequence) accurately in the presence of interfering substances which could be present in the samples.
Working range	The analyte concentration interval over which the method provides results with an acceptable uncertainty. In this concentration range, the relationship between response and concentration is continuous, reproducible and linear after suitable data transformation.
Accuracy	The closeness of agreement between a measurement result produced by the method for the analyte in a certain sample and the accepted reference value of that analyte. Accuracy can be divided into two parts:
• Precision	Measure of the variability in independent measurement results obtained for the same sample under stipulated conditions. There are three different levels depending on the conditions: repeatability, intermediate precision and reproducibility.
• Trueness	The closeness of agreement between the mean of an infinite number of measurement results produced by the method for the analyte in a certain sample and the accepted reference value of that analyte.
Measurement uncertainty	Interval associated with a measurement result which expresses the range of values that can reasonably be attributed to the analyte being measured.
Limit of detection (LOD)	The lowest analyte concentration that can be distinguished from zero, with a specified level of confidence.
Limit of quantification (LOQ)	The lowest analyte concentration for which the method provides results with an acceptable uncertainty.
Robustness (or ruggedness)	Measure of the capacity of the method to remain unaffected by small, but deliberate variations in method parameters.

The descriptions given in this table are based on the definitions and explanations as provided in several guidance documents [15], [16], [17].

**Table 2:** Results of the accuracy assessment of the BCR-ABL ddPCR method performed by measuring the five highest concentrations levels of ERM-AD623.

CRM	$c_{sample,cert}\pm U_{cert}$ (cp/μL)	${\bar{c}}_{sample,meas}$ (cp/μL)	$bias_{rel}$ (%)	$s_{repeat,rel}$ (%)	$s_{run,rel}$ (%)	$u_{precision,rel}$ (%)
AD623a	(1.08 ± 0.13) × 10^6^	0.97 × 10^6^	−10.2	4.7	1.4	1.1
AD623b	(1.08 ± 0.11) × 10^5^	0.93 × 10^5^	−13.8	5.6	5.3	3.2
AD623c	(1.03 ± 0.10) × 10^4^	0.94 × 10^4^	−9.0	4.8	2.7	1.8
AD623d	(1.02 ± 0.09) × 10^3^	0.93 × 10^3^	−8.5	7.7	0*	1.2
AD623e	(1.04 ± 0.10) × 10^2^	0.97 × 10^2^	−7.0	7.3	2.0	1.6

$c_{sample,cert}$: certified sample copy number concentration,$U_{cert}$: expanded uncertainty of the certified copy number concentration, ${\bar{c}}_{sample,meas}$: average measured sample copy number concentration, $bias_{rel}$: relative bias between the certified value and the measured value, $s_{repeat,rel}$: relative repeatability, $s_{run,rel}$: relative run-to-run variation, $u_{precision,rel}$: relative standard uncertainty related to the precision, *: $MS_{betweenrun}$ < $MS_{withinrun}$.

**Table 3:** Results of the experiment to assess the uncertainty contribution of the threshold setting.

$c_{PCR,exp}$(cp/μL)	Average number of positive droplets/measurement	Average number of negative droplets/measurement	Average number of rain droplets/measurement	$s_{thres}$(%)
5400	18046	414	95.9	3.0
2575	16005	2530	39.0	0.5
255	3488	16248	14.3	0.3
26	397	19457	4.1	0.8
2.5	38	19708	3.6	7.2

$c_{PCR,exp}$: the expected PCR copy number concentration based on the certified values of ERM-AD623, $s_{thres,rel}$: relative standard deviation associated with the threshold setting

**Table 4:** Results of the robustness test on the BCR-ABL ddPCR method investigating the effect of minor deviations in the primers/probe concentrations and annealing temperature.

Sample	Annealing temperature (°C)	Primers/probe concentration in PCR mix (nM)	${\bar{c}}_{PCR,meas}$ average ± STD (cp/μL)
ERM-AD623d	60	300/200	279 ± 5
	60	330/220	273 ± 4
	60	270/180	285 ± 8
ERM-AD623c	59	300/200	2946 ± 54
	60	300/200	2954 ± 35
	61	300/200	2948 ± 68

${\bar{c}}_{PCR,meas}$: the average measured PCR copy number concentration.
