# Supplementary material for: Validation of a digital PCR method for quantification of DNA copy number concentrations by using a certified reference material
Source: Biomol Detect Quantif. 2016 Aug 30;9:29–39. doi: 10.1016/j.bdq.2016.08.002 (PMC5007884; doi:10.1016/j.bdq.2016.08.002)
Supplement: Supplementary file 1 [file mmc1.docx]

**Supplementary data to the publication: "Validation of a digital PCR method for quantification of DNA copy number concentrations by using a certified reference material."**

**Table 1: Primers and probes used for the BCR-ABL and ABL ddPCR method.**

| **PCR target** | **Primer/ probe** | **Sequence** | **Concentration in PCR mix (nM)** | **Amplicon size (bp)** |
| --- | --- | --- | --- | --- |
| BCR-ABL | Forward primer | 5'-TCCGCTGACCATCAATAAGGA-3' | 300 | 149 |
|  | Reverse primer | 5'-CACTCAGACCCTGAGGCTCAA-3' | 300 |  |
|  | Probe | 5'-(6-VIC)-CCCTTCAGCGGCCAGTAG CATCTGA-(MGB)-3' | 200 |  |
| ABL | Forward primer | 5'-TGGAGATAACACTCTAAGCATAAC TAAAGGT-3' | 300 | 122 |
|  | Reverse primer | 5'-GATGTAGTTGCTTGGGACCCA-3' | 300 |  |
|  | Probe | 5'-(6-FAM)-CCATTTTTGGTTTGGGCTT CACACCATT-(TAMRA)-3' | 200 |  |

**Table 2: PCR protocol for both the BCR-ABL and the ABL ddPCR method**

| Step | Time  (s) | Temperature (°C) | Ramp rate  (°C/s) | Number of cycles |
| --- | --- | --- | --- | --- |
| Enzyme activation | 600 | 95 | 2.5 | 1 |
| Denaturation | 30 | 94 | 2.5 | 45 |
| Annealing/Extension | 60 | 60 | 2.5 |  |
| Enzyme deactivation | 600 | 98 | 2.5 | 1 |
| Hold | forever | 4 | 2.5 | 1 |

| **Table 3: dMIQE checklist for authors, reviewers and editors.**  **All essential information (E) must be submitted with the manuscript. Desirable information (D) should be submitted if possible.** | | |
| --- | --- | --- |
| **ITEM TO CHECK** | **IMPORTANCE** | **Comments** |
| **EXPERIMENTAL DESIGN** |  |  |
| Definition of experimental and control groups | **E** | Not applicable |
| Number within each group | **E** | Not applicable |
| Assay carried out by core lab or investigator's lab? | D | Not applicable |
| Power analysis | D | Not applicable |
| **SAMPLE** |  |  |
| Description | **E** | Included in manuscript |
| Volume or mass of sample processed | **E** | Not applicable |
| Microdissection or macrodissection | **E** | Not applicable |
| Processing procedure | **E** | Not applicable |
| If frozen - how and how quickly? | **E** | Not applicable |
| If fixed - with what, how quickly? | **E** | Not applicable |
| Sample storage conditions and duration (especially for FFPE samples) | **E** | Not applicable |
| **NUCLEIC ACID EXTRACTION** |  |  |
| Quantification - instrument/method | **E** | Not applicable |
| Storage conditions: temperature, concentration, duration, buffer | **E** | Not applicable |
| DNA or RNA quantification | **E** | Not applicable |
| Quality/integrity-instrument/method; e.g. RIN/RQI and trace or 3’:5’ | **E** | Not applicable |
| Template structural information | **E** | Not applicable |
| Template modification (digestion, sonication, pre-amplification etc.) | **E** | Not applicable |
| Template treatment (initial heating or chemical denaturation) | **E** | Not applicable |
| Inhibition dilution or spike; | **E** | Not applicable |
| DNA contamination assessment of RNA sample | **E** | Not applicable |
| Details of DNase treatment where performed | **E** | Not applicable |
| Manufacturer of reagents used and catalogue number | D | Not applicable |
| Storage of nucleic acid: temperature, concentration, duration, buffer | **E** | Not applicable |
| **REVERSE TRANSCRIPTION (If necessary)** |  |  |
| cDNA priming method + concentration | **E** | Not applicable |
| One or two step protocol | **E** | Not applicable |
| Amount of RNA used per reaction | **E** | Not applicable |
| Detailed reaction components and conditions | **E** | Not applicable |
| RT efficiency | D | Not applicable |
| Estimated copies measured with and without addition of RT* | D | Not applicable |
| Manufacturer of reagents used and catalogue number | D | Not applicable |
| Reaction volume (for two step reverse transcription reaction) | D | Not applicable |
| Storage of cDNA: temperature, concentration, duration, buffer | D | Not applicable |
| **dPCR TARGET INFORMATION** |  |  |
| Sequence accession number | **E** | Information already published [20,21] |
| Location of amplicon | D | Information already published [20,21] |
| Amplicon length | **E** | Included in manuscript |
| In silico specificity screen (BLAST, etc) | **E** | Information already published [20,21] |
| Pseudogenes, retropseudogenes or other homologs? | D | Not included in the manuscript |
| Sequence alignment | D | Not included in the manuscript |
| Secondary structure analysis of amplicon and GC content | D | Not included in the manuscript |
| Location of each primer by exon or intron (if applicable) | **E** | Not applicable |
| Where appropriate, which splice variants are targeted? | **E** | Not applicable |
| **dPCR OLIGONUCLEOTIDES** |  |  |
| Primer sequences and/or amplicon context sequence** | **E** | Included in manuscript |
| RTPrimerDB Identification Number | D | Not included in the manuscript |
| Probe sequences** | D | Included in manuscript |
| Location and identity of any modifications | **E** | Included in manuscript |
| Manufacturer of oligonucleotides | D | Included in manuscript |
| Purification method | D | Included in manuscript |
| **dPCR PROTOCOL** |  |  |
| Complete reaction conditions | **E** | Included in manuscript |
| Reaction volume and amount of RNA/cDNA/DNA | **E** | Included in manuscript |
| Primer, (probe), Mg++ and dNTP concentrations | **E** | Included in manuscript |
| Polymerase identity and concentration | **E** | Included in manuscript |
| Buffer/kit Catalogue No and manufacturer | **E** | Included in manuscript |
| Exact chemical constitution of the buffer | D | Included in manuscript |
| Additives (SYBR Green I, DMSO, etc.) | **E** | Included in manuscript |
| Plates/tubes Catalogue No and manufacturer | D | Included in manuscript |
| Complete thermocycling parameters | **E** | Included in manuscript |
| Reaction setup | D | Included in manuscript |
| Gravimetric or volumetric dilutions (manual/robotic) | D | Included in manuscript |
| Total PCR reaction volume prepared | D | Included in manuscript |
| Partition number | **E** | Included in manuscript |
| Individual partition volume | **E** | Included in manuscript |
| Total volume of the partitions measured (effective reaction size) | **E** | Included in manuscript |
| Partition volume variance/standard deviation | D | Included in manuscript |
| Comprehensive details and appropriate use of controls | **E** | Included in manuscript |
| Manufacturer of dPCR instrument | **E** | Included in manuscript |
| **dPCR VALIDATION** |  |  |
| Optimisation data for the assay | D | Included in manuscript |
| Specificity (when measuring rare mutations, pathogen sequences etc.) | **E** | Included in manuscript |
| Limit of detection of calibration control | D | Included in manuscript |
| If multiplexing, comparison with singleplex assays | **E** | Not applicable |
| **DATA ANALYSIS** |  |  |
| Average copies per partition (λ or equivalent ) | **E** | Included in manuscript |
| dPCR analysis program (source, version) | **E** | Included in manuscript |
| Outlier identification and disposition | **E** | Included in manuscript |
| Results of NTCs | **E** | Included in manuscript |
| Examples of positive(s) and negative experimental results as supplemental data | **E** | Description of results included in the manuscript |
| Where appropriate, justification of number and choice of reference genes | **E** | Not applicable |
| Where appropriate, description of normalisation method | **E** | Not applicable |
| Number and concordance of biological replicates | D | Not applicable |
| Number and stage (RT or qPCR) of technical replicates | **E** | Included in manuscript |
| Repeatability (intra-assay variation) | **E** | Included in manuscript |
| Reproducibility (inter-assay/user/lab etc. variation ) | D | Included in manuscript |
| Experimental variance or confidence interval*** | **E** | Included in manuscript |
| Statistical methods used for analysis | **E** | Included in manuscript |
| Data submission using RDML | D | Not included in the manuscript |
| * Assessing the absence of DNA using a no RT assay (or where RT has been inactivated) is essential when first extracting RNA. Once the sample has been validated as DNA-free, inclusion of a no-RT control is desirable, but no longer essential. | | |
| ** Disclosure of the primer and probe sequence is highly desirable and strongly encouraged. However, since not all commercial pre-designed assay vendors provide this information when it is not available assay context sequences must be submitted (Bustin et al. Clin Chem. 2011 Jun;57(6):919-21.) | | |
| *** When single dPCR experiments are performed, the variation due to counting error alone should be calculated from the binomial (or suitable equivalent) distribution. | | |


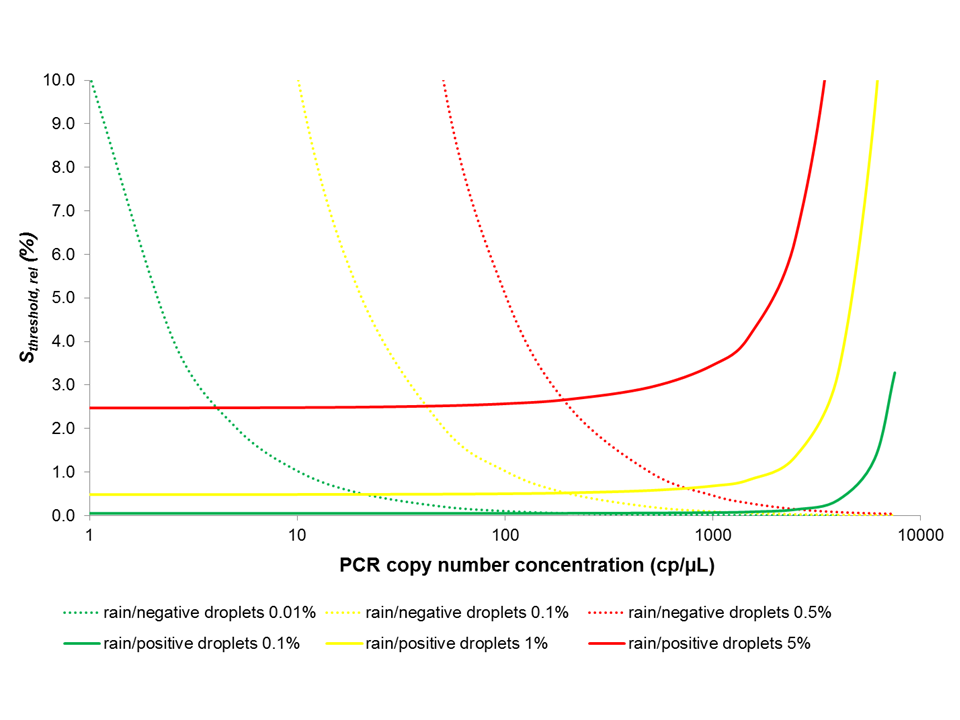


**Figure 1: The theoretical simulation of the maximum uncertainty related the threshold setting (**$\boldsymbol{s}_{\boldsymbol{threshold,rel}}$**) for the ddPCR system.**

The $s_{threshold,rel}$ was calculated as the relative standard deviation among the results obtained with four completely different approaches for the classification of the rain droplets:

- Half of the rain droplets are considered positive and the other half are considered negative (simulating a threshold at the midpoint between the positive and the negative cluster)
- All rain droplets are considered positive (simulating a low threshold placed at the upper boundary of the negative droplet cluster)
- All rain droplets are considered negative (simulating a high threshold placed at the lower boundary of the positive droplet cluster)
- All rain droplets were excluded and not considered as accepted droplets
